# Community resource centres to improve the health of women and children in informal settlements in Mumbai: a cluster-randomised, controlled trial

**DOI:** 10.1016/S2214-109X(16)30363-1

**Published:** 2017-02-11

**Authors:** Neena Shah More, Sushmita Das, Ujwala Bapat, Glyn Alcock, Shreya Manjrekar, Vikas Kamble, Rijuta Sawant, Sushma Shende, Nayreen Daruwalla, Shanti Pantvaidya, David Osrin

**Affiliations:** a Society for Nutrition, Education and Health Action, Mumbai, India; b UCL Institute for Global Health, London, UK

## Abstract

**Background:**

Around 105 million people in India will be living in informal settlements by 2017. We investigated the effects of local resource centres delivering integrated activities to improve women's and children's health in urban informal settlements.

**Methods:**

In a cluster-randomised controlled trial in 40 clusters, each containing around 600 households, 20 were randomly allocated to have a resource centre (intervention group) and 20 no centre (control group). Community organisers in the intervention centres addressed maternal and neonatal health, child health and nutrition, reproductive health, and prevention of violence against women and children through home visits, group meetings, day care, community events, service provision, and liaison. The primary endpoints were met need for family planning in women aged 15–49 years, proportion of children aged 12–23 months fully immunised, and proportion of children younger than 5 years with anthropometric wasting. Census interviews with women aged 15–49 years were done before and 2 years after the intervention was implemented. The primary intention-to-treat analysis compared cluster allocation groups after the intervention. We also analysed the per-protocol population (all women with data from both censuses) and assessed cluster-level changes. This study is registered with ISRCTN, number ISRCTN56183183, and Clinical Trials Registry of India, number CTRI/2012/09/003004.

**Findings:**

12 614 households were allocated to the intervention and 12 239 to control. Postintervention data were available for 8271 women and 5371 children younger than 5 years in the intervention group, and 7965 women and 5180 children in the control group. Met need for family planning was greater in the intervention clusters than in the control clusters (odds ratio [OR] 1·31, 95% CI 1·11–1·53). The proportions of fully immunised children were similar in the intervention and control groups in the intention-to-treat analysis (OR 1·30, 95% CI 0·84–2·01), but were greater in the intervention group when assessed per protocol (1·73, 1·05–2·86). Childhood wasting did not differ between groups (OR 0·92, 95% CI 0·75–1·12), although improvement was seen at the cluster level in the intervention group (p=0·020).

**Interpretation:**

This community resource model seems feasible and replicable and may be protocolised for expansion.

**Funding:**

Wellcome Trust, CRY, Cipla.

## Introduction

More than 377 million people live in India's 7933 urban areas,^1^, ^2^ of which 53 house more than 1 million people each. Three mega-cities, Mumbai, Delhi, and Kolkata, house more than 10 million people each. Two-thirds of census towns include informal settlements (slums)^3^ that are characterised by overcrowding, insubstantial housing, insufficient water and sanitation, lack of tenure, and hazardous locations.^4^, ^5^ There will be around 105 million people living in informal settlements by 2017.^2^ India's National Urban Health Mission aims to facilitate equitable access to quality health care through an improved public health system, partnerships, and community-based mechanisms. Three tiers of provision are envisaged: secondary and tertiary institutions, urban health centres, and community outreach to informal settlements and other vulnerable groups.^6^ This structure frames a context of pluralistic health care. For example, Mumbai's population of 12·4 million^7^ is served by a pyramid of municipal tertiary hospitals, peripheral hospitals, maternity homes, and health posts. Additionally, there are charitable institutions and a wealth of private care providers (from specialist hospitals to unqualified practitioners), the latter of which are responsible for around 75% of outpatient consultations.^8^

Non-governmental organisations are important to public–private partnerships,^9^ in which they contribute services traditionally provided by the public sector,^10^ alone or in collaboration,^11^ and develop models for adoption by the public sector.^6^ The Society for Nutrition, Education and Health Action (SNEHA) is a non-governmental organisation whose programmes address priority issues that have emerged from 16 years of work with women and children in informal settlements: maternal and neonatal health, sexual and reproductive health, childhood nutrition, and prevention of violence against women and children. We wanted to integrate these activities in a model that could be useful to the National Urban Health Mission of India and other city governments in achieving a commitment to health in informal settlements.^12^ After a large trial focused on neonatal survival,^13^ we believed that integration of the programme in the community was appropriate because of the multiple health issues faced by women and children, and that communities were more likely to respond to an intervention with a physical presence and service delivery.

**Research in context**

**Evidence before this study**

We searched PubMed for articles published up to Oct 1, 2016, addressing health-care interventions in urban slums worldwide. We used English search terms, but placed no restriction on the language of retrieved articles. We used the combined search expression “(slum OR “informal settlement”) AND (healthcare OR “health care”) AND (provision OR delivery OR program$ OR project)”. We screened 1481 article titles, including 389 limited to Asia and 175 limited to India, from which we identified 48 relevant abstracts. We found no completed or published trial of a model of provision of integrated health care for informal settlement populations, although models with some similarities are operational in Delhi and Chennai in India and in Bangladesh.

**Added value of this study**

We showed that a community resource centre model for women's and children's health was feasible and potentially replicable and incurred low cost in informal settlements. The intervention could be implemented by a non-governmental organisation in collaboration with public sector and civil society institutions. It was possible to measure population health outcomes, with effects seen after only 2 years of operation.

**Implications of all the available evidence**

This clearly defined model for integrated community-based health intervention in informal settlements merits adaptation and assessment in other contexts, particularly in Asia and Africa.

We conceived a model that included service provision, outreach, and community mobilisation activities, with a visible presence in SNEHA centres. The evidence base for this type of approach is limited. Data synthesis identified 17 reviews of interventions to improve health in informal settlements, including physical upgrading of the built environment, improvements in water and sanitation, infectious disease control, prevention of burns, and cash transfers.^14^ Ten randomised, controlled trials of health-promotion interventions included interventions by community health workers to improve handwashing and nutrition and reduce the risks of burns, poisoning, injuries, and HIV infection.^14^ Relevant Cochrane reviews included the effects on health of strategies to upgrade slums^15^ and a planned review of childhood nutritional interventions.^16^

We found no completed trial of health-care provision to people living in informal settlements, but we identified three regional initiatives that informed our model. In Delhi, the non-governmental Asha Community Health and Development Society has, since 1988, developed a programme of land rights advocacy, education, savings and loans, health care and diagnosis, community health volunteers and groups, and campaigns on these issues.^17^ In Bangladesh, the non-governmental BRAC MANOSHI programme has provided interventions for maternal, neonatal, and child health in urban informal settlements since 2007. It involves community health workers, birth attendants working in local delivery centres, and settlement committees.^18^, ^19^ In an informal settlement in Chennai, India, the Sahishnatha Trust delivered an integrated intervention that addressed water and sanitation and provided female link workers, weekly health clinics, self-help groups, and community campaigns.^20^

In this study, we implemented the SNEHA centre model and assessed the effects across a range of outcomes representative of women's and children's health. The study protocol has been published.^21^

## Methods

### Study design and setting

We used a parallel-group, phased,^22^ cluster-randomised, controlled trial design, and did censuses before and after the intervention. About 41% of Mumbai's households are in informal settlements.^3^ We did this trial in two of 24 municipal wards, each with a population of around 700 000. The wards were chosen because they had the lowest Human Development Indices (M East ward 0·05 and L ward 0·29).^23^ The numbers of informal settlements in these wards have grown over the past 20 years. Most have surfaced roads, electricity supplies, and schools. They are low-lying and susceptible to flooding, and some adjoin the city's largest solid-waste dump. About 65% of the settlement populations are made up of migrants from Uttar Pradesh, 10% from Bihar, and 15% from Maharashtra, the state in which Mumbai is located. Work includes unskilled labour (40%), skilled artisanal work (27%), and transport (14%). At the start of the study, the study area was served by nine municipal health posts, one urban health centre, one maternity home that provided antenatal care, immunisations, and family planning, but was underused, and a tertiary public hospital that took about 20 min to reach from the study area by public transport. Additionally, 42 Anganwadi centres, run by the Indian Government Integrated Child Development Services (ICDS) programme, provided maternal and child health and nutrition services. Many private practitioners, with a range of qualifications, tailor their services to the local economy. We identified 35 private providers in the intervention clusters alone.

Before the trial, data for Mumbai slums were available from the National Family Health Survey (NFHS) in 2005–06,^8^ which showed that met need for family planning was 55% (6% for spacing and 49% for limiting of pregnancies). Full immunisation with BCG, diphtheria, pertussis, and tetanus ([DPT] three doses), polio, and measles vaccines was 69% in children aged 12–23 months. Wasting was seen in 16% of children younger than 5 years, and stunting in 47%.^8^ Violence against women is common in India.^24^ Estimates of lifetime prevalence are 29% (range 2–99) for domestic physical violence, 12% (0–75) for sexual violence, and 30% (4–56) for multiple forms of violence.^25^ However, violence is under-reported, and the incidence has been cited as 54·8 assaults per 100 000 women in the general population.^26^

We estimated that about 60 sizeable non-governmental organisations were working in informal settlements in Mumbai. Some, including the Society for Promotion of Area Resource Centres, Akanksha Foundation, Apnalaya, DoorstepSchool, and Pratham, had run community resource centres with varied purposes: education, vocational training, recreational activities, centres for people with disabilities, family counselling, collective savings and loan disbursement, physical space for community interaction, and health clinics. Some organisations, including Apnalaya, Stree Hitkarni, the Committed Communities Development Trust, Alert, and Navjeevan, had focused on community health. Their resource centres were staffed by a mix of volunteers and salaried teams.

The trial was approved by the Multi-Institutional Ethics Committee of the Anusandhan Trust, Mumbai, India, in sequential reviews: formative research (February, 2011), cluster vulnerability (May, 2011), the preintervention census (August, 2011), and the intervention and assessments (January, 2012). It was also approved by the University College London Research Ethics Committee, London, UK, in January, 2012 (reference 3546/001).

### Participants

**Figure 1:**
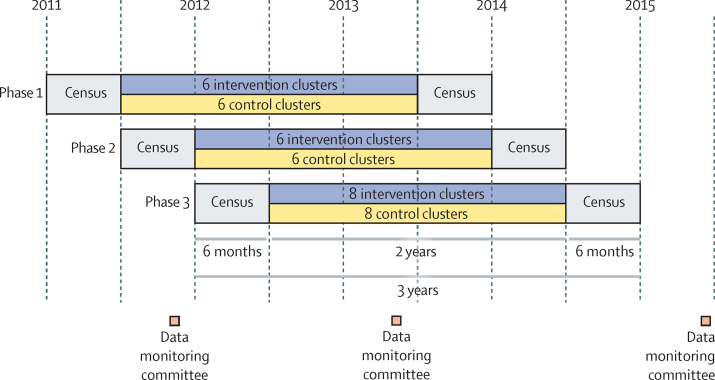
Trial design

The resource centres targeted women of reproductive age and children younger than 5 years, but any other residents living in an allocated cluster were free to participate in activities and access services. Two censuses were done, one before and one 2 years after implementation of the intervention (figure 1). Interviewees were ever-married women aged 15–49 years.

We obtained consent at the cluster and individual levels^27^ and no monetary compensation was given. Cluster consent was provided by cluster gatekeepers, who were identified with a standard protocol from among participants in community engagement activities who others in the area judged appropriate to speak for them on health issues. Receipt of the intervention was at individual discretion. The right to withdraw at any time was implicit. Respondents in both censuses were given standard information about the trial and the procedures for anonymising data.

### Ethical considerations

We identified no specific risks of harm to individuals or the community associated with community resource centres themselves. If, however, women and children were identified by data collectors as being malnourished, concerned about family planning or birth in an institution, or being survivors of domestic violence, interviewers had a duty of care to act within personal and organisational abilities. We followed guidelines on the reporting of violence, which included obtaining consent from the survivor and explaining the available interventions. Campaigns and group sessions were used to explain to survivors that they could disclose abuse and access support at any time they felt ready. For individual or family problems, community organisers followed organisational support procedures and protocols, provided information packs on local sources of help, and aided in arranging consultations. The study ethics committee mandated protocols for training, information, and action across a range of issues. As the intervention was part of our service delivery programme, we judged it to carry minimum risk over 2 years and did not specify stopping rules.

### Randomisation and masking

**Figure 2:**
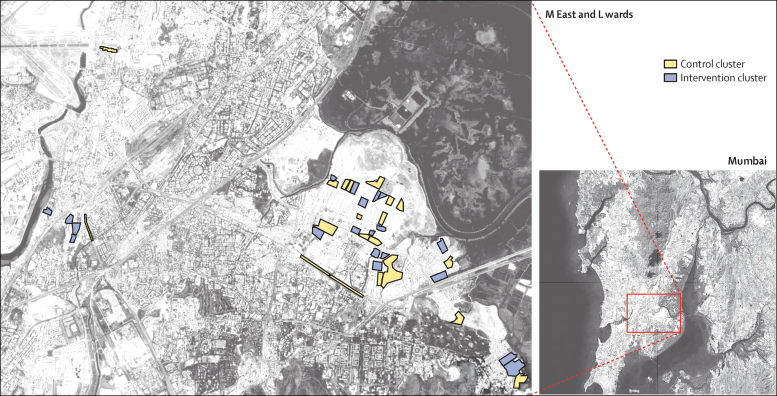
Locations of clusters in the M East and L wards of Mumbai included in randomisation

The sample frame included 159 clusters of around 600 households. We identified informal settlements through local knowledge and information from the Municipal Corporation of Greater Mumbai, the Tata Institute of Social Sciences, and non-governmental organisations. We excluded areas that had been involved in a previous trial^13^ and divided large settlements into clusters along obvious physical boundaries, using geographical distribution to minimise contamination. After assessment of health vulnerability in all clusters in the sample frame with a rapid assessment tool,^28^ we included the 40 with the lowest scores in the study (figure 2). On July 25, 2011, SD and DO used an online randomisation generator to randomly assign these clusters, in blocks of 12, 12, and 16, to the intervention group or the control group, with intervals of 6 months between the allocation of each block to create three implementation phases (figure 1). Project staff were unaware of allocation when they collected consent and did the preintervention census. Because of the nature of the intervention, the implementation team and the field investigators who did the postintervention census were aware of allocation. However, to keep familiarity with residents to a minimum, a new team was recruited to do the postintervention census.

### Procedures

A SNEHA centre was set up in each intervention cluster in rented premises. Each centre employed three full-time, salaried community organisers who were educated to at least the ninth grade and had similar socioeconomic backgrounds to potential beneficiaries. Each organiser was responsible for around a third of the households in the cluster. We engaged the community organisers to integrate our themes of reproductive, maternal, and neonatal health, child health and nutrition, and prevention of violence against women and children into the community services. They were equipped with technical knowledge in these areas and with communication and negotiation skills through 1 month of training followed by regular supervision and follow-up visits by SNEHA staff and invited experts. They made home visits, organised group meetings, day care for malnourished children, and community events, provided services, and liaised with existing systems (panel 1). All activities were logged via a smartphone-based reporting system created from open-source software (CommCare, Dimagi, Cambridge, MA, USA), including information about the families with whom they worked.

The censuses were done by two teams of six interviewers and a supervisor who clarified cluster boundaries and mapped and numbered households. Interviews with eligible women were used to obtain information on numbers of household members, duration of residence, assets and amenities, housing fabric, faith, maternity history, and family planning. Women who had been pregnant in the preceding 2 years were asked about antenatal care, birth location, outcome, and infant feeding. Information on immunisations and use of ICDS was collected for children younger than 5 years. Additionally, anthropometric characteristics were recorded for children younger than 5 years on designated days at the end of each cluster census. Length of children younger than 2 years was measured with a Rollameter accurate to 1 mm, with an assistant holding the child's head. Height of children aged 2 years and older was measured with a Leicester stadiometer accurate to 1 mm, at the end of expiration, with feet together against the backboard, back straight, and head in the Frankfort plane. Weight was measured with Seca 385 electronic scales accurate to 1 g. Training for data collectors was repeated on four occasions, for which the indicative technical errors of measurement for height were 0·14%, 0·38%, 0·6%, and 0·5%.

Interview data were collected on smartphones with an open-source tool from Open Data Kit (Seattle, WA, USA) running in Google Android (versions 3.0–4.4 [Honeycomb to Kitkat]). 5% of interviews selected at random were observed by a supervisor. The interview system included automatic skips and validation constraints. Encrypted electronic data were transferred to a secure Open Data Kit Aggregate cloud repository on a password-protected Google Appspot (Google App Engine). Data were checked after download for errors in key fields, and monitoring summaries were produced through do-files written in Stata version 12. Each week, after all interviews from that week were numbered, 50 records (20–25% of interviews) were extracted at random, printed on spreadsheets, and rechecked in the field by a supervisor. After the interviewees' names had been removed, access to data was restricted to the data manager and data analysts.

### Outcomes

Outcomes were assessed on the basis of the two censuses done before and after 2 years of the intervention being implemented (panel 2). We assessed three primary outcomes: met need for family planning in women aged 15–49 years; the proportion of children aged 12–23 months who were fully immunised (BCG, DPT [three doses], polio, hepatitis B virus, and measles); and the proportion of children younger than 5 years who had anthropometric wasting. We also assessed seven secondary outcomes: number of consultations for violence against women or children; the proportion of home births in the preceding year; the proportion of pregnancies in the preceding 2 years in women younger than 20 years; the proportion of children younger than 5 years with anthropometric stunting; the proportion of children younger than 5 years with anthropometric underweight; the proportion of children born in the preceding 2 years who received ICDS; and the proportion of children achieving WHO Infant and Young Child Feeding core indicators (panel 2).^30^

### Statistical analysis

For our sample size calculations, we assumed that we would be able to achieve two treatment groups consisting of unmatched clusters of roughly equal size and with similar *k* values (coefficient of variation of true proportions between clusters).^31^ On the basis of the data from the preintervention census, when around 400 women were interviewed per cluster, and the estimates that per cluster around 80 children would have been born from around 80 pregnancies in the previous 2 years and that there would be roughly 120 children aged 2–5 years, we calculated that interviewing 350 women and measuring the weights and heights of 150 children younger than 5 years per cluster after the intervention would provide 80% power to detect a 5% increase in met need for family planning, a 13% increase in full immunisation, and a 4% reduction in anthropometric wasting with a 5% significance threshold.

Wealth was described by asset scores. We used a principal components analysis to derive weights for scores.^32^, ^33^

We generated anthropometric *Z* scores from the 2006 WHO growth standards and the ZSCORE06 module in Stata/IC (version 13.1).^34^ Outliers were removed so that the *Z* scores ranged from −6 to 6 for height for age, from −5 to 5 for weight for length or height, and from −6 to 5 for weight for age.^35^ We derived binary variables describing wasting, stunting, and underweight, with the threshold for all set at 2 SD below the median WHO value.

We compared frequencies and proportions of demographic, socioeconomic, and environmental descriptors and of primary and secondary outcomes before the intervention in the two allocation groups, and report odds ratios (ORs) with 95% CIs. The primary intention-to-treat analysis involved a series of logistic regression models, including a variable for the outcome of interest, a dummy variable for allocation, and a random effect for cluster (quadrature assumptions were met). The allocation groups were generally balanced and we did not introduce additional covariates. Likelihood ratio tests showed no evidence of effect modification by implementation phase and, therefore, models did not include an interaction term.^36^

We did two additional analyses. First, because migration rates were high, we did a per-protocol analysis of women who had participated in both censuses (ie, those who had potentially been exposed to the intervention for the full 2 years). Second, because weight for length or height differed between groups before the intervention, we did a cluster-level analysis of anthropometric changes in mean *Z* scores and the proportions of children with wasting between censuses. We did the same for uptake of family planning and proportions fully immunised at 12–23 months, and applied *t* tests to the normally distributed changes. Data to develop a classification of met need were unavailable in the baseline census and, therefore, we defined use of modern contraception as female or male terminal methods, oral contraceptive pill, intrauterine device, hormone implant or injection, condom, or diaphragm.

We used the RE-AIM framework to describe delivery and uptake of the intervention.^37^ 34 components were reported, classified under five general criteria:^37^, ^38^ reach (the proportion of the target population who participated in the intervention, according to the census data), efficacy (based on trial endpoint findings), adoption (use of the intervention in allocated clusters), implementation (the degree to which the intervention was delivered as intended, assessed by programme monitoring), and maintenance of the intervention after the implementation period. Scores allocated were within the potential range of 0·0–1·0. The overall score was the product of the five criteria scores. We estimated the cost of the intervention from organisational finance records. Cost codes covered salaries, communication, and conveyance for human resources at all levels up to programme director, set-up and running costs for centres, equipment and consumables, costs of training, meetings, events and campaigns, internal monitoring costs, and administrative overheads.

A data monitoring committee met twice during the trial and once after the second census (figure 1). Following DAMOCLES guidelines,^39^ at the first meeting in May, 2012, the committee considered the protocol, sealed the analysis plan, and reviewed the findings of the preintervention census from the 12 clusters in phase 1 of implementation. In the second meeting, in December, 2013, data from all three implementation phases were assessed; no changes to the protocol were recommended. In the third meeting, in January, 2016, the final data were reviewed and ancillary analyses were recommended, leading to an addendum to the published protocol. After the first meeting, we removed receipt of the Janani Suraksha Yojana birth incentive as a secondary indicator because we were unable to affect its use at the institutional level. At the time of protocol development, we planned to use the London Measure of Unplanned Pregnancy^40^ as a measure of family planning. In 2012, revised guidelines for estimating unmet need for family planning were released,^41^ which we used instead. This study is registered with ISRCTN, number ISRCTN56183183, and Clinical Trials Registry of India, number CTRI/2012/09/003004.

### Role of the funding source

The funder of the study had no role in study design, data collection, data analysis, data interpretation, or writing of the report. The corresponding author had full access to all the data in the study and had final responsibility for the decision to submit for publication.

## Results

**Figure 3:**
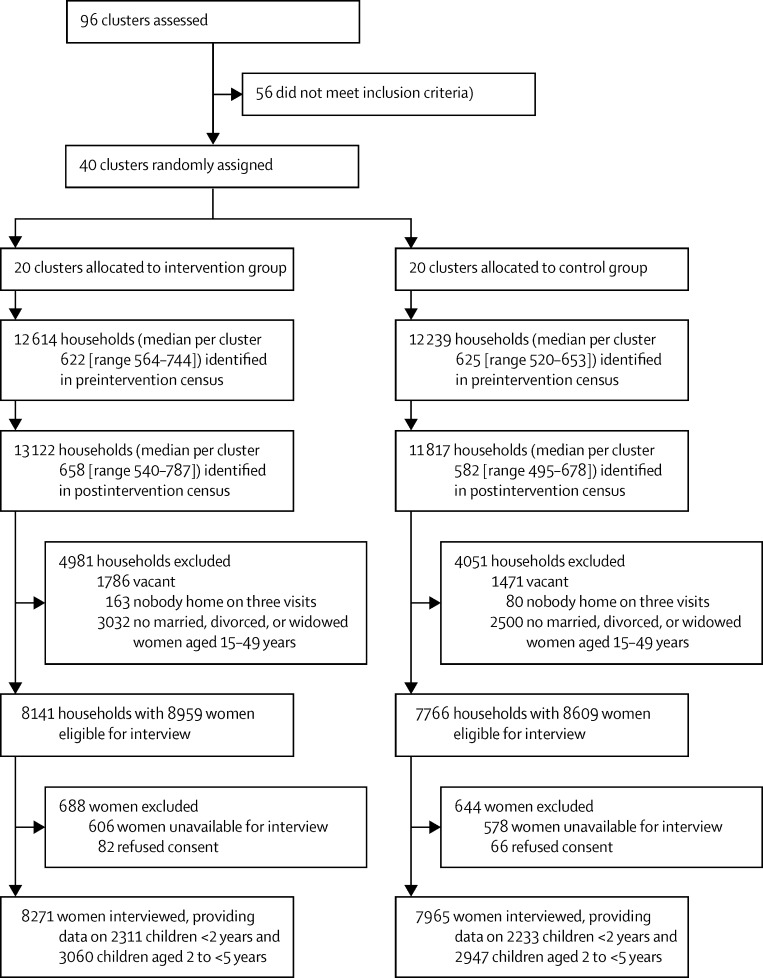
Trial profile

The preintervention census started in August, 2011, and was completed in January, 2013, and the postintervention census began in February, 2014, and was completed in September, 2015 (figure 1). The preintervention census identified 24 853 households and the postintervention census identified 24 939 (figure 3). Of the postintervention households, 15 907 (63·8%) were home to 17 568 eligible women, of whom 16 236 (92·4%) were interviewed. Information was provided for 10 551 children younger than 5 years.

Characteristics of respondents were similar in the intervention and control groups in the preintervention census (table 1). We saw substantial environmental improvements in the postintervention census versus the preintervention census. The number of home owners was unchanged, but increases were seen for robust housing fabric (10 908 [75%] of 14 474 houses *vs* 8399 [59%] of 14 293), households with a private tap providing drinking water (3483 [24%] *vs* 2533 [18%]), access to a community tapstand for drinking water (9372 [65%] *vs* 2553 [18%]), and homes with private toilets (2604 [18%] *vs* 1612 [11%]), and a decrease was seen in the number of households buying drinking water from tankers (1619 [11%] *vs* 9207 [64%]).

Before the intervention, about 30% of women said that they were using modern methods of contraception, of which female terminal methods were the most common, 64% of children aged 12–23 months were fully immunised, and around 16% of children younger than 5 years showed anthropometric wasting and 47% showed stunting (table 2). The mean *Z* scores for weight for length or height were −0·92 (SD 1·15) in the control group and −1·06 (1·12) in the intervention group, and for height for age were −1·82 (1·65) and −1·70 (1·67), respectively. Only 5% of children aged 6–23 months met the requirements for minimum acceptable diet (table 2). In the intention-to-treat analysis after the intervention, met need for family planning was greater in the intervention than the control group for both spacing and limiting of pregnancies (table 3). Adjustment for maternal age and parity increased the likelihood of overall met need (1·35, 1·14–1·60). Values for full immunisation and wasting did not differ between allocation groups, although children aged 12–23 months in the intervention group were more likely to have immunisation cards than those in the control group (table 3). For children younger than 5 years, the mean *Z* scores for weight for length or height were −0·90 (SD 1·00) in the control group and −0·88 (1·02) in the intervention group.

**Figure 4:**
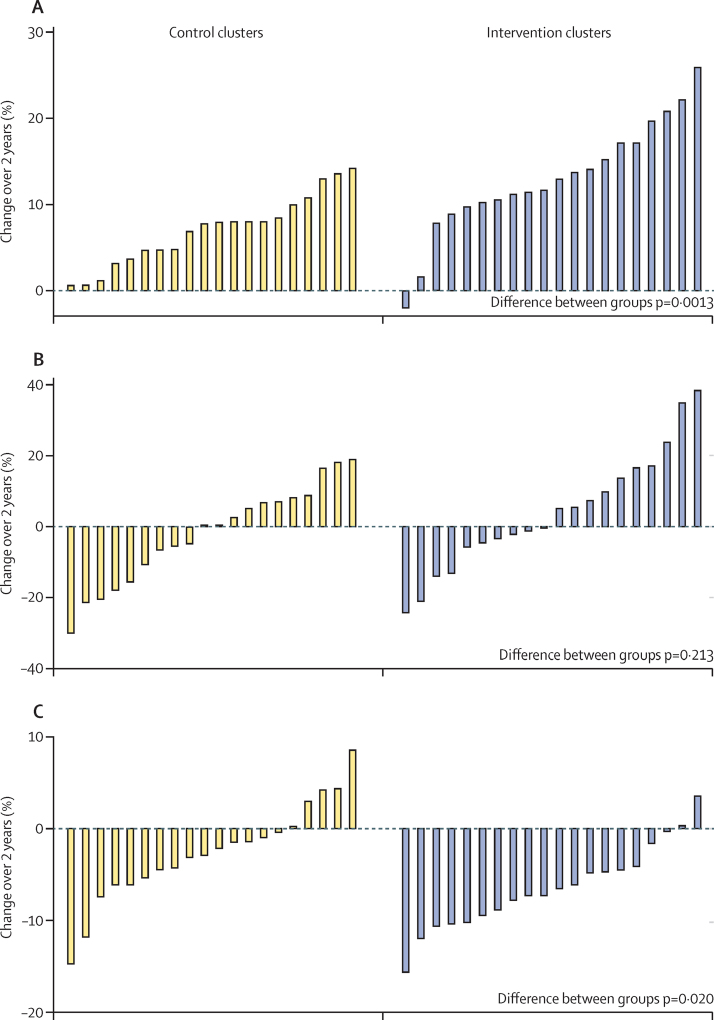
Changes after intervention in use of modern contraception, full immunisation of children aged 12–23 months, and anthropometric wasting in children younger than 5 years in the per-protocol analysis (A) Use of modern contraception, defined as female or male terminal methods, oral contraceptive pill, intrauterine device, hormone implant or injection, condoms, or diaphragm. (B) Full immunisation, defined as BCG, diphtheria, pertussis, and tetanus (three doses), polio, hepatitis B virus (three doses), and measles. (C) Anthropometric wasting, defined as values more than 2 SD below the median WHO value for weight for length or height for age and sex.

The per-protocol analysis of the primary endpoint included 5838 households, 5830 women, and 3529 children younger than 5 years (2647 households, 2645 women, and 1560 children in the control group, and 3191 households, 3185 women, and 1969 children in the intervention group). Thus, 40% of all households and 36% of all women participated in both censuses. After the intervention, use of modern family planning methods had increased by a mean of 7·0% in the control group and 13·1% in the intervention group, leading to a significant difference between groups (figure 4). Full immunisation in children aged 12–23 months changed by a mean of −1·9% in the control group and 4·2% in the intervention group but the difference between groups was not significant (figure 4). Weight for height *Z* score increased by a mean of 0·03 in the control group and by a mean of 0·19 in the intervention group (p=0·013). The proportion of children with anthropometric wasting decreased by a mean of 2·5% and 6·4%, respectively, leading to a significant difference between groups (figure 4).

Services for survivors of violence reported 314 consultations in intervention clusters. The proportions of births at home, childhood stunting and underweight, and uptake of ICDS for children aged 0–23 months did not differ between allocation groups. Decreases in *Z* scores for height for age were seen in both groups (mean −1·84 [SD 1·41] in the control group and −1·86 [1·37] in the intervention group) and weight for age (–1·70 [1·13] and −1·68 [1·13]). Feeding exclusively with breastmilk up to age 6 months and achieving minimum dietary diversity in children aged 6–23 months were increased in the intervention group compared with in the control group after the intervention (OR 1·54, 95% CI 1·02–2·33 and 1·48, 1·01–2·17).

In the intervention group, 84% of women knew of the SNEHA centre in their cluster, 79% recalled monthly visits from a community organiser, 39% had participated in SNEHA centre activities, and 88% of women with children younger than 5 years reported had received a service (table 4). In the control group, fewer than 1% of residents reported awareness of or participating in any similar activities. Uptake of municipal or non-governmental services did not differ between groups.

In the summary of the intervention process done with the adapted RE-AIM framework (appendix), we estimated scores of 0·8 for reach, 0·7 for effectiveness, 1·0 for adoption, 0·8 of implementation, and 0·8 for maintenance. The overall product score was 0·36. We estimated that the cost of the intervention was INR694 000 per SNEHA centre per year (£7300, US$10 340) or INR231 350 (£2435, $3450) per 1000 general population.

## Discussion

Our integrated intervention, which combined home visits, group work, some service provision, and liaison, was delivered in some of the poorest of Mumbai's informal settlements by a non-governmental organisation. We saw clear improvements in various indicators of women's and children's health, including met need for family planning and full immunisation of children. The effect on immunisation was, however, seen only when women had been exposed to the intervention for 2 years (the per-protocol population). Effects on childhood malnutrition were not evident from the analysis of anthropometric wasting, but could be inferred from additional analyses (figure 4).

In the NFHS of 2015–16 (NFHS-4),^42^ unmet need for family planning was estimated to be 14% for Mumbai. In our control group after the intervention, however, unmet need was 22%, which suggests a difference between informal settlements and the city as a whole. Intervention might, therefore, be particularly important in informal settlements. The NFHS-4 findings showed that 46% of children aged 12–23 months in Mumbai were fully immunised.^42^ The comparable proportion in this study in the postintervention census (excluding hepatitis B and measles) was higher at 69%, as were the values for individual vaccinations (BCG 95% in our intervention group *vs* 88% in the NFHS-4; DPT 82% *vs* 51%; and hepatitis B 79% *vs* 46%). Anthropometric wasting in children was lower in our intervention group than in the NFHS-4 (12% *vs* 26%), although the proportion for stunting was greater (47% *vs* 23%). The values for neonates breastfed within 1 h of birth were similar (47% after our intervention *vs* 50% in NFHS-4) and more children in our intervention group were receiving an adequate diet at age 6–23 months (13% *vs* 5%).

Our intervention and control groups were generally similar, with high coverage and fidelity to planned activities and negligible contamination. Some of our outcomes were proxies for longer-term effects. For example, the most widely used forms of contraception were female terminal methods (44% of women in the control group and 35% in the intervention group) and condoms (21% and 30%). The increase in met need for family planning was largely due to escalating condom use. Changes in spacing and limiting of pregnancies will only be possible to assess in the longer term, particularly given the possibility of best behaviour bias in the intervention clusters. Similarly, although an end itself, an aim of immunisation is to reduce cause-specific morbidity and mortality, but the effects will only be possible to assess over the long term.

The effects of the intervention on full immunisation and anthropometric wasting in children were limited, for which we offer several possible explanations. First, exposure to the intervention at the individual level might have been insufficient. Although coverage was high, population turnover was around 30% annually, and 2 years was a short time in which to assess the effects of the intervention and to consolidate community involvement. We will continue to assess the effects over the coming years. Young children moving into the clusters from elsewhere without primary immunisation also meant that achieving full immunisation in all children was unlikely, despite the efforts of community organisers. Second, although we found no evidence of contamination of control clusters by the intervention, government schemes and the activities of municipal and non-governmental providers might have improved health in control clusters. Nevertheless, we saw no evidence of increased use of other providers in the control clusters (table 4). Additionally, births in institutions rather than at home have become the norm. We had hoped that the intervention would increase the use of municipal health care and ICDS and concurrently strengthen ICDS,^43^ but we saw no indication of these effects. A third possibility is secular change. Environmental indicators improved substantially during the period of the intervention. The proportion of homes made with robust fabric increased by 16% and that of households with private toilets by 7%. Purchase of drinking water from tankers fell by 53%. Although anthropometric wasting in children was reduced after the intervention in the intervention group (which monitoring data from day-care centres suggest was causal), improvement from low preintervention levels was also seen in control areas. We tested several hypotheses to explain improvements in the control group (appendix p 3), but saw no differential changes between control and intervention areas in housing quality, water supply, economic poverty, schooling, migration numbers, or state of origin.

Informal settlements have different cultural, structural, and legal statuses from formal settlements, but we think that our findings are generalisable to established informal settlements that have some amenities and high annual turnover. The range of issues that community organisers had to address led to rapport with beneficiaries, but challenged their ability to focus on our primary indicators. Training needs were extensive, daily work involved multiple home visits and group facilitation, and response to requests from beneficiaries was time-consuming. For example, a whole day might be spent in accompanying one woman to support her health-care needs or in responding to a domestic violence incident. A possible option is to prioritise activities that address risk over general activities, for instance by decreasing the frequency of growth monitoring for children who are doing well.

In pursuit of the 11th United Nations Sustainable Development Goal, city governments in India and elsewhere are seeking guidance on the use of resources to improve health in informal settlements. We believe the evidence from this trial suggests effectiveness with a community resource centre model. Certainly, activities may be protocolised, making the model feasible and replicable, and we are currently expanding the catchment area to achieve economy of scale.

## Figures and Tables

**Table 1:** Characteristics of households and respondents in the preintervention census

		**Control group**	**Intervention group**
**Households**			
All		12 614 (100%)	12 239 (100%)
Median (IQR) households per cluster		622 (609–649)	625 (584–637)
Median (IQR) household size		5 (5–6)	5 (5–5)
Number of homes		7317 (100%)	6976 (100%)
Home owned		4359 (60%)	4189 (60%)
Family had ration card		4660 (64%)	4453 (64%)
Housing fabric			
	Robust (*pucca*)	4071 (56%)	4328 (62%)
	Partly robust (*semi-pucca*)	1922 (26%)	1807 (26%)
	Temporary (*kaccha*)	1324 (18%)	841 (12%)
Electricity supply			
	None	8 (<1%)	8 (<1%)
	Metered (family pay bill)	4720 (64%)	4673 (67%)
	Family pay landlord	568 (8%)	674 (10%)
	Other	2021 (28%)	1621 (23%)
Drinking water source			
	Private tap	1091 (15%)	1442 (21%)
	Community tap stand	1241 (17%)	1312 (19%)
	Purchased from tanker or in containers	4985 (68%)	4222 (60%)
Toilet			
	Private	807 (11%)	805 (12%)
	Public	6382 (87%)	6170 (88%)
	No toilet facility	128 (2%)	1 (<1%)
Flooring			
	Dirt, sand, mud	481 (7%)	447 (6%)
	Concrete, brick, tiles	6836 (93%)	6529 (94%)
Asset index quintile			
	1 (poorest)	1798 (25%)	1518 (22%)
	2	1247 (17%)	1157 (17%)
	3	1462 (20%)	1414 (20%)
	4	1446 (20%)	1440 (21%)
	5 (least poor)	1364 (19%)	1447 (21%)
**Women respondents**			
Number of respondents		8227 (100%)	7947 (100%)
Age (years)			
	15–19	244 (3%)	241 (3%)
	20–29	3425 (42%)	3300 (41%)
	30–39	2874 (35%)	2760 (35%)
	40–49	1684 (20%)	1646 (21%)
Religion			
	Muslim	6231 (76%)	6591 (83%)
	Hindu	1967 (24%)	1291 (16%)
	Other	29 (<1%)	65 (<1%)
Education			
	None	3292 (40%)	2943 (37%)
	Primary	644 (8%)	565 (7%)
	Secondary	3846 (47%)	3928 (49%)
	Higher	441 (5%)	504 (6%)
	Missing	4 (<1%)	7 (<1%)
Length of residence in Mumbai (years)			
	<1	459 (6%)	461 (6%)
	1–5	1189 (14%)	1080 (14%)
	6–10	875 (11%)	870 (11%)
	>10	2096 (25%)	1977 (25%)
	Lifelong	2700 (33%)	2588 (33%)
	Missing	908 (11%)	971 (12%)
Always lived in current home		3344 (41%)	3309 (42%)
Parity			
	0	836 (10%)	817 (10%)
	1	1218 (15%)	1199 (15%)
	2	1637 (20%)	1588 (20%)
	3	1577 (19%)	1518 (19%)
	≥4	2958 (36%)	2824 (36%)
	Missing	1 (<1%)	1 (<1%)
Pregnancy in previous 2 years		2225 (27%)	2117 (27%)

**Table 2:** Outcome indicators in the preintervention census

				**Control group**	**Intervention group**
**Primary outcomes**					
Number of women respondents				8227 (100%)	7947 (100%)
Number using family planning*				2414 (29%)	2294 (29%)
Family planning method					
	Female terminal			1333 (55%)	1207 (53%)
	Oral contraceptive pill			466 (19%)	419 (18%)
	Condom			293 (12%)	337 (15%)
	Intrauterine contraceptive device			202 (8%)	214 (9%)
	Other			120 (5%)	117 (5%)
Immunisation in children aged 12–23 months†					
	Number of children			1014 (100%)	945 (100%)
	Fully immunised			637 (63%)	613 (65%)
	BCG			935 (92%)	880 (93%)
	DPT and polio				
			Dose 1	881 (87%)	839 (89%)
			Dose 2	832 (82%)	798 (84%)
			Dose 3	769 (76%)	757 (80%)
	Hepatitis B virus			731 (72%)	679 (72%)
	Measles			674 (66%)	672 (71%)
Anthropometric wasting in children <5 years‡				586 (15%) of 3881§	640 (18%) of 3550§
**Secondary outcomes**					
Deliveries to women in the previous 2 years					
	All			2022 (100%)	1905 (100%)
	Adolescent pregnancy			286 (14%)	264 (14%)
	Home delivery			297 (15%)	287 (15%)
	Institutional delivery			1719 (85%)	1588 (83%)
		Public institutional delivery		1102 (64%)	1017 (64%)
			Received public institutional delivery incentive	555 (50%)	550 (54%)
	Unknown			6 (<1%)	30 (2%)
Anthropometric stunting and underweight in children aged 0–59 months					
	Stunting			1851 (48%) of 3861§	1632 (46%) of 3541§
	Underweight			1541 (39%) of 3902§	1472 (41%) of 3576§
Infant and young child feeding indicators					
	Early initiation of breastfeeding			999 (48%)	841 (44%)
	Exclusive breastfeeding (<6 months)			357 (62%)	309 (62%)
	Continued breastfeeding at 1 year (12–15 months)			270 (73%)	245 (72%)
	Introduction of solid, semisolid, or soft foods (6–8 months)			90 (35%)	120 (47%)
	Minimum dietary diversity (6–23 months)			192 (13%)	186 (13%)
	Minimum meal frequency (6–23 months)			646 (43%)	630 (43%)
	Minimum acceptable diet (6–23 months)			67 (4%)	71 (5%)
	Consumption of iron-rich foods (6–23 months)			205 (14%)	238 (16%)
Use of ICDS services					
	Children <5 years eligible			5057 (100%)	4767 (100%)
	Used ICDS			466 (9%)	508 (11%)
	Food supplements almost daily			373 (7%)	391 (8%)
	Health check-ups at least once per month			163 (3%)	179 (4%)
	Regular early childhood development intervention			306 (6%)	340 (7%)
	Weight measured at least once per 3 months			333 (7%)	349 (7%)

DPT=diphtheria, pertussis, and tetanus. ICDS=Government of India Integrated Child Development Services.

* Intracluster correlation coefficient 0·011.

† Intracluster correlation coefficient 0·066.

‡ Intracluster correlation coefficient 0·008.

§ Height or weight measurements were not taken for some children.

**Table 3:** Outcomes in the postintervention census

			**Control group**	**Intervention group**	**Intention-to-treat OR (95% CI)**	**Per-protocol OR (95% CI)**
**Primary outcomes**						
Met need for family planning among women aged 15–49 years			3134 (78%) of 4028	3439 (82%) of 4184	1·31 (1·11–1·53)	1·37 (1·07–1·75)
Full immunisation among children aged 12–23 months			708 (62%) of 1143	751 (68%) of 1108	1·30 (0·84–2·01)	1·73 (1·05–2·86)
Anthropometric wasting in children <5 years			580 (13%) of 4608	530 (12%) of 4570	0·92 (0·75–1·12)	0·88 (0·66–1·18)
**Secondary outcomes**						
Home births in previous 2 years			276 (12%) of 2266	270 (12%) of 2202	1·14 (0·69–1·86)	1·25 (0·65–2·41)
Adolescent pregnancies in previous 2 years in women <20 years			210 (9%) of 2266	199 (9%) of 2202	0·96 (0·69–1·33)	0·95 (0·55–1·65)
Anthropometric stunting in children <5 years			2105 (46%) of 4610	2146 (47%) of 4573	1·03 (0·85–1·25)	1·08 (0·84–1·40)
Anthropometric underweight in children <5 years			1766 (38%) of 4610	1791 (39%) of 4573	1·03 (0·88–1·21)	1·06 (0·88–1·29)
Use of ICDS by children aged 0–23 months			25 (1%) of 2243	18 (<1%) of 2177	1·16 (0·72–1·89)	0·89 (0·53–1·51)
**Infant and young child feeding indicators**						
Early initiation of breastfeeding			975 (44%) of 2198	1005 (47%) of 2146	1·10 (0·58–2·07)	1·11 (0·55–2·25)
Exclusive breastfeeding (<6 months)			310 (56%) of 554	329 (66%) of 504	1·54 (1·02–2·33)	1·95 (1·02–3·76)
Continued breastfeeding at 1 year (12–15 months)			318 (77%) of 411	288 (78%) of 368	1·05 (0·75–1·48)	1·58 (0·89–2·82)
Introduction of solid, semisolid, or soft foods (6–8 months)			154 (52%) of 297	180 (58%) of 308	1·27 (0·75–2·15)	0·91 (0·44–1·88)
Minimum dietary diversity (6–23 months)			274 (16%) of 1751	374 (22%) of 1718	1·48 (1·01–2·17)	1·54 (0·99–2·39)
Minimum meal frequency (6–23 months)			1048 (60%) of 1751	1142 (66%) of 1718	1·26 (0·91–1·75)	1·21 (0·83–1·78)
Minimum acceptable diet (6–23 months)			160 (9%) of 1751	218 (13%) of 1718	1·39 (0·89–2·17)	1·58 (0·94–2·65)
Consumption of iron-rich foods (6–23 months)			282 (16%) of 1751	291 (17%) of 1718	1·05 (0·76–1·45)	1·25 (0·86–1·80)
**Further information on primary and secondary outcomes**						
Family planning						
	Use of modern contraception to space pregnancies		442 (14%) of 3134	598 (17%) of 3439	1·29 (1·06–1·58)	1·23 (0·95–1·60)
	Use of modern contraception to limit pregnancies		1036 (33%) of 3134	1433 (42%) of 3439	1·44 (1·21–1·71)	1·15 (0·91–1·45)
	Planned pregnancy in previous 2 years (London Measure of Unplanned Pregnancy)		1678 (65%) of 2575	1799 (71%) of 2532	1·20 (0·84–1·73)	1·33 (0.86–2·04)
Reported intimate partner violence in previous 1 year			1052 (14%) of 7705	556 (7%) of 7484	0·85 (0·44–1·64)	0·90 (0·46–1·72)
Immunisation for children aged 12–23 months						
	Immunisation card		551 (48%) of 1143	645 (58%) of 1108	1·52 (1·14–2·02)	1·58 (1·04–2·40)
	Fully immunised on immunisation card		395 (72%) of 1143	497 (77%) of 1108	1·30 (0·84–2·01)	1·73 (1·05–2·01)
	BCG		1077 (94%) of 1143	1054 (95%) of 1108	1·06 (0·61–1·87)	0·84 (0·24–3·01)
DPT and polio						
		Dose 1	991 (87%) of 1143	1000 (90%) of 1108	1·41 (0·78–2·55)	1·98 (0·87–4·49)
		Dose 2	905 (79%) of 1143	947 (85%) of 1108	1·52 (0·93–2·50)	2·80 (1·49–5·24)
		Dose 3	846 (74%) of 1143	905 (82%) of 1108	1·56 (1·03–2·38)	2·44 (1·42–4·20)
Hepatitis B virus						
		Dose 1	964 (84%) of 1143	973 (88%) of 1108	1·42 (0·80–2·54)	2·34 (1·03–5·29)
		Dose 2	883 (77%) of 1143	923 (83%) of 1108	1·49 (0·91–2·43)	2·36 (1·29–4·35)
		Dose 3	829 (73%) of 1143	874 (79%) of 1108	1·44 (0·93–2·25)	2·06 (1·24–3·43)
	Measles		735 (64%) of 1143	786 (71%) of 1108	1·34 (0·87–2·06)	1·99 (1·19–3·34)
Immunisation for children aged 24–59 months						
	Immunisation card		1077 (35%) of 3060	1713 (58%) of 2947	1·38 (0·91–2·10)	1·15 (0·76–1·74)
	BCG		2882 (94%) of 3060	2824 (96%) of 2947	1·25 (0·74–2·10)	1·36 (0·51–3·66)
	DPT and polio					
		Dose 1	2684 (88%) of 3060	2685 (91%) of 2947	1·33 (0·86–2·05)	1·54 (0·87–2·75)
		Dose 2	2550 (83%) of 3060	2588 (88%) of 2947	1·32 (0·88–1·98)	1·72 (1·00–2·96)
		Dose 3	2475 (81%) of 3060	2517 (85%) of 2947	1·27 (0·87–1·86)	1·57 (0·95–2·60)
	Hepatitis B virus					
		Dose 1	2634 (86%) of 3060	2610 (89%) of 2947	1·16 (0·76–1·77)	1·23 (0·69–2·19)
		Dose 2	2510 (82%) of 3060	2515 (85%) of 2947	1·18 (0·79–1·76)	1·36 (0·81–2·27)
		Dose 3	2436 (80%) of 3060	2442 (83%) of 2947	1·12 (0·77–1·63)	1·21 (0·73–2·01)
	Measles		2245 (73%) of 3060	2323 (79%) of 2947	1·25 (0·83–1·88)	1·39 (0·83–2·33)
Anthropometric malnutrition in children aged 0–59 months						
	Severe acute		93 (2%) of 4608	68 (1%) of 4570	0·73 (0·48–1·13)	0·54 (0·27–1·09)
	Moderate acute		487 (11%) of 4608	462 (10%) of 4570	0·95 (0·78–1·17)	0·96 (0·71–1·29)
Use of government ICDS by children aged 0–24 months						
	Food supplements almost daily		13 (<1%) of 2243	10 (<1%) of 2177	1·16 (0·55–2·45)	1·73 (0·71–4·24)
	Health check-ups at least once per month		8 (<1%) of 2243	9 (<1%) of 2177	0·92 (0·49–1·73)	1·31 (0·64–2·68)
	Regular early childhood development intervention		3 (<1%) of 2243	5 (<1%) of 2177	1·40 (0·51–3·83)	1·71 (0·53–5·56)
	Weight measured at least once per 3 months		12 (<1) of 2243	8 (<1%) of 2177	1·73 (0·88–3·39)	2·39 (1·10–5·20)

OR=odds ratio. ICDS=Government of India Integrated Child Development Services. DPT=diphtheria, pertussis, and tetanus.

**Table 4:** Intervention coverage, contamination, and substitution in the postintervention census

		**Control group**	**Intervention group**	**p value**
**Women aged 15–49 years**				
Number of women		8271(100%)	7965(100%)	N/A
Aware of local SNEHA centre		75 (<1%)	6661 (84%)	<0·0001
Aware of services offered by SNEHA centre		41 (<1%)	6299 (79%)	<0·0001
	Growth monitoring	38 (<1%)	6170 (77%)	N/A
	Immunisation	15 (<1%)	2437 (31%)	N/A
	Child health checks	32 (<1%)	4802 (60%)	N/A
	Nutrition education	18 (<1%)	3688 (46%)	N/A
	Family planning	24 (<1%)	2896 (36%)	N/A
	Counselling for violence against women and girls	12 (<1%)	1947 (24%)	N/A
Visited by community organiser		14 (<1%)	6981 (88%)	<0·0001
	Visited at least monthly	12 (<1%)	6291 (79%)	N/A
Participated in SNEHA centre activities		6 (<1%)	3108 (39%)	<0·0001
	Group meetings	5 (<1%)	2958 (37%)	N/A
	Parents' meetings	1 (<1%)	704 (9%)	N/A
	Recipe workshops	2 (<1%)	848 (11%)	N/A
Received municipal services in previous year		2711 (33%)	2760 (35%)	0·708
	Antenatal care	717 (9%)	737 (9%)	N/A
	Delivery care	759 (9%)	675 (8%)	N/A
	Family planning	247 (3%)	156 (2%)	N/A
	Immunisation	1209 (15%)	1231 (15%)	N/A
	Health camp	1582 (19%)	1626 (20%)	N/A
Received other NGO services in previous year		259 (3%)	206 (3%)	0·493
	Growth monitoring	717 (26%)	737 (27%)	N/A
	Delivery care	53 (<1%)	3 (<1%)	N/A
	Immunisation	18 (<1%)	1 (<1%)	N/A
	Child health check	29 (<1%)	7 (<1%)	N/A
	Family planning	103 (1%)	121 (2%)	N/A
**Women with children <5 years**				
Number of women		3800(100%)	3777(100%)	N/A
Received SNEHA centre service		16 (<1%)	3332 (88%)	<0·0001
	Growth monitoring	36 (<1%)	3299 (87%)	N/A
	Immunisation	2 (<1%)	869 (23%)	N/A
	Child health checks	9 (<1%)	2357 (62%)	N/A
	Nutrition education	7 (<1%)	1506 (40%)	N/A
	Family planning	2 (<1%)	714 (19%)	N/A
	Counselling for violence against women and girls	0	69 (2%)	N/A
Aware of day-care centre for children		12 (<1%)	1803 (48%)	<0·0001
Aware of services offered by day-care centre		9 (<1%)	1574 (42%)	0·256
	Day care	7 (<1%)	983 (26%)	N/A
	Growth monitoring	9 (<1%)	1458 (39%)	N/A
	Immunisation	5 (<1%)	780 (21%)	N/A
	Supplementary food	7 (<1%)	1321 (35%)	N/A
	Child health checks	8 (<1%)	1162 (31%)	N/A
	Play and learning	4 (<1%)	741 (20%)	N/A
Have used day-care centre		4 (<1%)	608 (16%)	0·894
	Child admitted	1 (<1%)	443 (12%)	N/A
	Growth monitoring	4 (<1%)	579 (15%)	N/A
	Immunisation	0	237 (6%)	N/A
	Supplementary food	2 (<1%)	528 (14%)	N/A
	Child health check	3 (<1%)	466 (12%)	N/A
	Play and learning	1 (<1%)	331 (9%)	N/A

N/A=not applicable. SNEHA=Society for Nutrition, Education and Health Action. NGO=non-governmental organisation.

## Panel 1 Study intervention

**Inception microplanning**

We facilitated a series of participatory learning and action and resource-mapping exercises.^19^ 5-day cycles involved community volunteers building rapport with each other and the cluster community, and facilitating understanding of community resources, patterns of health care, and local aspirations. Activities involved community members, front-line workers from other organisations, and resource agencies, and the findings were disseminated within communities and allied systems. Programme staff also established contact with public sector providers and local non-governmental organisations and held stakeholder workshops to streamline referrals.

**Communication emphasis**

• Maternal and neonatal health: registration of pregnancy, antenatal care, referral for women at risk of pregnancy-related disorders, nutrition counselling, institutional delivery, essential neonatal care (eg, resuscitation, warmth, early and frequent breastfeeding, keeping with mother, cleanliness, and prompt identification of illness), and referral for obstetric and neonatal danger signs.
• Child health and nutrition: feeding of infants and young children, immunisation, treatment of severe acute malnutrition, and seeking care for illness.
• Sexual and reproductive health: family planning, adolescent sexual and reproductive health, and life skills.
• Prevention of violence against women and children: disclosure, reporting, community-based crisis intervention, and support.

**Activities**

• Home visits: each community organiser visited ten homes per day in which they identified and provided information for family health needs, referred people to appropriate institutions, provided help with access, and negotiated with family members to facilitate appropriate choices. Home visits were open-ended, but health needs, providing appropriate information, and accessing services for women of reproductive age and their children were prioritised.
• Group meetings: community organisers facilitated daily group meetings with between five and 15 people, including pregnant women, mothers of malnourished children, men, and adolescents, in the SNEHA centre, an open space, or a member's home to address women's and children's health concerns through information exchange, peer learning, and discussion, based on established participatory group approaches.^13^
• Day care for malnourished children: a day-care centre annex to the SNEHA centre was established in each cluster, staffed by a teacher and an aide trained in early childhood development activities by Mumbai Mobile Creches. Children with acute malnutrition (anthropometric wasting), classified as weight for length or height more than 2 SD below the WHO median for age and sex for moderate or more than 3 SD for severe, were identified and enrolled by community organisers and were screened by a clinician. Children with severe acute malnutrition were prescribed local medical nutrition therapy for 56 days with ready-to-use therapeutic food. Those with moderate acute malnutrition were prescribed four meals per day, including milk, egg, fruit, lunch from home, and the take-home ration provided by the Integrated Child Development Services. Children attending day-care centres were seen weekly by a SNEHA clinician and were referred to a paediatrician if they did not improve or they developed illness. Other day-care activities included early child development stimulation, rest, attention to hygiene, and a monthly parents' meeting.
• Community events: events aimed at increasing awareness and creating an environment conducive to women's and children's health. These included puppet shows, street plays, rallies, games, competitions, “flash mobs”, and cooking demonstrations. Traditional events, such as the Godbharai baby shower and Ushtavan initiation of complementary feeding, were complemented by events celebrating improvements in child health and nutrition that provided opportunities to discuss health behaviours. International events, such as Breastfeeding and Nutrition Week and 16 Days Activism against Violence, were highlighted.
• Service provision: counsellors were available to support survivors of physical, emotional, sexual, or economic violence by intimate or non-intimate partners. Women reporting violence were offered participation in an extensive support and response programme that included crisis intervention with counselling, psychotherapy, and family intervention, and support with police complaints and legal redress. Visiting clinicians who rotated weekly across SNEHA centres could provide some over-the-counter allopathic medications (anthelmintics, oral rehydration salts, antimalarials, eye and ear drops, oral antimicrobials, oral analgesics, and micronutrient supplements) and make referrals to paediatricians. Community organisers provided family planning directly (condoms and oral contraceptive pills) or by referral to the Family Planning Association of India under a formal memorandum.
• Liaison: we worked with the Municipal Corporation of Greater Mumbai and the Indian Government Child Development Services to improve communication with communities and strengthen outreach and uptake. Regular meetings were held with front-line and supervisory groups to share data, plan, and deliver activities, such as outreach immunisation camps, growth monitoring, follow-up on referrals, and supplementary nutrition provided in Indian Government Child Development Services Anganwadi centres. Periodic meetings with senior officials focused on sharing concerns and developing strategies to improve access to and quality of public services.

SNEHA=Society for Nutrition, Education and Health Action.

## Panel 2 Outcome definitions

**Primary outcomes**

• Met need for family planning among women aged 15–49 years, derived from the unmet need ascribed to married, widowed, separated, or divorced women who were pregnant but said that they did not want to be, who said they did not want any more children but were not using contraception, or who were using family planning but not with modern methods
• Proportion of children aged 12–23 months fully immunised (BCG, diphtheria, pertussis, and tetanus [three doses], polio, hepatitis B virus [three doses], and measles^29^)
• Proportion of children younger than 5 years with anthropometric wasting, defined as weight for length or height more than 2 SD below the median WHO value for age and sex

**Secondary outcomes**

• Number of consultations for violence against women or children, including physical (slapping, arm twisting, pushing, punching, kicking, choking, or use of implements or weapons), emotional (jealousy, accusations of infidelity, prevention of association with friends or family, aggressive monitoring of whereabouts, lack of trust with money, humiliation in front of others, threats of violence, or insults), or sexual (forced sex, coercion into undesired sexual acts)
• Proportion of home births in the preceding year
• Proportion of pregnancies in the preceding 2 years in women younger than 20 years
• Proportion of children younger than 5 years with anthropometric stunting
• Proportion of children younger than 5 years with anthropometric underweight
• Proportion of children born in the preceding 2 years who received services from Government of India Integrated Child Development Services (food supplements, health checks, early childhood development intervention, or measurement of weight)
• Proportion of children meeting Infant and Young Child Feeding core indicators: early initiation of breastfeeding, defined as breastfeeding within 1 h of birth; exclusive breastfeeding of children younger than 6 months, defined as receiving only breastmilk during the previous day; continued breastfeeding at 1 year, defined as children aged 12–15 months receiving breastmilk in the previous day; introduction of solid, semisolid, or soft foods in children, defined as children aged 6–8 months receiving such foods in the previous day; minimum dietary diversity, defined as children aged 6–23 months receiving foods from four or more food groups; minimum meal frequency, defined as breastfed and non-breastfed children aged 6–23 months receiving solid, semisolid, or soft foods (including milk feeds for non-breastfed children); minimum acceptable diet, defined as children aged 6–23 months receiving at least minimum dietary diversity and minimum meal frequency during the previous day in breastfed children and at least minimum dietary diversity (excluding milk feeds) and minimum meal frequency plus at least two milk feeds during the previous day in non-breastfed children; and consumption of iron-rich or iron-fortified foods in children aged 6–23 months^30^
