# Supplementary material for: Community resource centres to improve the health of women and children in informal settlements in Mumbai: a cluster-randomised, controlled trial
Source: Lancet Glob Health. 2017 Feb 11;5(3):e335–49. doi: 10.1016/S2214-109X(16)30363-1 (PMC5316560; doi:10.1016/S2214-109X(16)30363-1)
Supplement: Supplementary appendix [file mmc1.pdf]

THE LANCET
Global Health

Supplementary appendix

This appendix formed part of the original submission and has been peer reviewed. We post it as supplied by the authors.

Supplement to: More NS, Das S, Bapat U, et al. Community resource centres to improve the health of women and children in informal settlements in Mumbai: a cluster-randomised, controlled trial. *Lancet Glob Health* 2017; **5:** e335–49.

Webtable 1. RE-AIM framework criteria[35]

Criterion	Assessment	Score
**Reach**		**0.8**
Exclusion criteria (% excluded or characteristics)	Intervention targeted women aged 15-49 and children under five. Households absent such individuals were not visited regularly.<br>High inclusion	0.9
Percentage of individuals who participated, based on valid denominator	Summarised in Table 4. Quantitative census report of awareness and uptake of services<br>High participation	0.8
Characteristics of participants compared with nonparticipants	Summarised in Tables 1, 2, and 3<br>Quantitative census report of sociodemographic, healthcare, and outcome indices.<br>High similarity	0.9
Use of qualitative methods to understand recruitment	Summarised in process evaluation report.<br>High uptake	0.8
**Effectiveness**		**0.7**
Measure of primary outcome	Uptake of family planning: intention-to-treat increase: score 0.8<br>Child immunization: increases in sentinel categories: score 0.7<br>Child malnutrition: per-protocol improvements at cluster level: score 0.6<br>Moderate evidence	0.7
Measure of primary outcome relative to public health goal	Moderate evidence of improvement, but transient population context and limited increment.<br>Measurable, but not substantial	0.7
Measure of broader outcomes or use of multiple criteria (eg quality of life or potential negative outcome)	Multiple outcomes assessed. No evidence of negative outcomes. Quality of life not assessed.<br>Some improvement	0.7
Measure of robustness across subgroups (eg moderation analysis)	No differences in outcomes between intervention phase, socioeconomic groupings, religious groups.<br>Robust within context	0.8
Measure of short-term attrition (%) and differential rates by treatment group	Substantial attrition. Rate differential between intention-to-treat and per protocol groups.<br>High attrition	0.4
Use of qualitative methods to understand outcomes	Summarised in process evaluation report.<br>Good understanding of context and modifiers	0.7
**Adoption: setting level**		**1.0**
Setting exclusions (% or reasons or both)	No exclusions.<br>High inclusion	1.0
Percentage of settings approached that participate (valid denominator)	No refusals of 40 approached<br>High participation	1.0
Characteristics of settings participating (both comparison and intervention compared with either nonparticipants or some relevant resource data)	Summarised in Tables 1, 2, and 3<br>Quantitative census report of sociodemographic, healthcare, and outcome indices.<br>High similarity	1.0
Use of qualitative methods to understand setting level adoption	Summarised in process evaluation report.<br>Good understanding of context	1.0
**Adoption: staff level**		**1.0**
Staff exclusions (% or reasons or both)	None excluded<br>High inclusion	1.0
Percent of staff offered that participate	All staff participated<br>High participation	1.0
Characteristics of staff participants v nonparticipating staff or typical staff	All staff participated<br>No nonparticipants	1.0
Use of qualitative methods to understand staff participation or staff level adoption	Summarised in process evaluation report.<br>Human resource characteristics clear	1.0
**Adoption**		**1.0**
**Implementation**		**0.8**
Percent of perfect delivery or calls completed (eg fidelity)	Summarised in monitoring information system and Table 4.<br>High delivery quality	0.8
Adaptations made to intervention during study (not fidelity)	Adaptations between phases 1 and 2.<br>Minor changes to improve delivery	0.8
Cost of intervention: time	3 full-time community organisers per SNEHA Centre, 6-8 per supervisor, 1 manager<br>Moderate	0.8
Cost of intervention: money	SNEHA Centre rents: moderate cost<br>Materials and consumables: low cost<br>Human resource costs: moderate	0.8
Consistency of implementation across staff, time, settings, subgroups	Routines specified, monitored, and fed back in quality improvement cycle, including cross-team comparison.	0.8

Use of qualitative methods to understand implementation	High consistency<br>Summarised in process evaluation report.<br>Strong commitment to fidelity and aims	0.8
**Maintenance: individual**		**0.7**
Measure of primary outcome (with comparison with a public health goal) at >= 6 months follow-up after final treatment contact	Continued collection of monitoring data.	0.8
Measure of primary outcome at >= 6 months follow-up after final treatment contact	Continued collection of monitoring data.	0.8
Measure of broader outcomes (eg measure of quality of life or potential negative outcome) or use of multiple criteria at follow-up	Multiple indicators for follow-up.	0.6
Robustness data: something about subgroup effects over the long-term	Assumed from monitoring data, but not yet tested formally.	0.7
Measure of long-term attrition (%) and differential rates by patient characteristics or treatment condition	Assumed from monitoring data, but not yet tested formally.	0.5
Use of qualitative methods data to understand long-term effects	Assumed from monitoring data, but not yet tested formally.	0.6
**Maintenance: setting**		**0.9**
If program is still ongoing at >=6 months posttreatment follow-up	Program ongoing, with modifications to improve economy of scale and sustainability.<br>Sustained commitment to program	0.9
If and how program was adapted long-term (which elements retained after program completion)	Adaptation of childhood nutrition component.<br>Adaptation of catchment area to increase coverage per centre.<br>High evidence adaptation	0.8
Some measure or discussion of alignment to organization mission or sustainability of business model	Model conceived, tested, and modified as central to organizational mission and sustainability of business model.<br>High alignment	0.9
Use of qualitative methods data to understand setting level institutionalisation	To be collected as model rolls out to other institutions	
**Maintenance**		**0.8**

Webtable 2. Indicators hypothesised to explain improvement in childhood wasting in control clusters

	Level after intervention period (%)		Change over 2 years (%)	
**Indicator that might have improved more in control than in intervention areas**	**Control**	**Intervention**	**Control**	**Intervention**
Rented homes	42.8	42.9	2.4	2.7
Robust housing	73.0	78.7	18.0	17.2
Possession of ration card	57.6	58.8	-6.1	-4.6
Water supply at home or community tap	87.1	89.9	54.4	52.0
Private toilet	18.3	16.9	7.5	5.9
Asset index (mean)	0.03	0.07	0.07	0.05
Resident in Mumbai <2 y	10.3	10.1	1.3	0.2
Resident in home <2 y	29.8	29.7	-0.1	-0.6
No schooling	35.7	32.7	-3.6	-4.7
From Bihar, Haryana, Punjab, Uttar Pradesh	73.3	74.0	0.63	3.86
Hindu	24.4	16.3	-0.4	-0.6
Household residents (mean)	5.3	5.4	-0.1	-0.1

Observations and interviews in two control clusters in which wasting in children 0-59 months had decreased most

Cluster A

Overall area

The area looked a little better-off than the other areas, with almost all houses of robust fabrication, covered drains, and paved roads. It was relatively clean, although there were some garbage dumps in open areas surrounding the clusters. Children looked clean and well dressed. The community organiser said that it had been like this since before the baseline census, and that the environment had not improved over the last two years.

Water supply

The area was on a hill and water taps, owned privately by residents, were mostly located low on the hill. The community was busy with men, women and children filling cans of water, ferrying them back to their houses higher on the hill to empty into drums, and coming back for another round. These people said that the situation had not changed in the last few years.

Integrated Child Development Services (ICDS)

There were two Anganwadis (ICDS centres) in the cluster and one on the boundary with another area. All three Anganwadis were closed when we visited them in the late morning, but two were open by 12:30. Anganwadi workers said that they had done a lot of awareness raising and that people’s habits were improving, but local residents said that the Anganwadis were barely open for an hour a day, for rapid distribution of food after which children were sent home.

Doctors' opinions

We met with two popular doctors who had different views on the situation. One said that women had become more competitive about their children's health and there was demand for supplementary formula feeding. He said that levels of awareness had increased and that parents were more vigilant. The other doctor said that levels of alcohol use had fallen as a result of closure of liquor shops in the area, resulting in reduced domestic problems. He said he received fewer complaints about domestic quarrels and their sequelae.

Residents’ opinions

Women in the cluster said that they did not perceive any change in the environment or people's habits that would have improved child health and nutrition. They said that there were many new shops in the area and children ate a lot of food bought from outside the home. They said that this junk food was useless and was not nutritious like homemade food. They seemed dissatisfied with the Anganwadis, but did not know what to do about it.

Cluster B

Overall area

The area was next to a creek and marshland covered in garbage. Research team members said that many new huts had been built since the baseline census, and some were visible towards the ends of lanes leading into the marsh. The lanes mostly had covered drains and were fairly clean.

Water supply

Water supply was via a few privately owned connections, for which most people had to pay the owners. The houses generally had semi-robust ground floors with sheet metal rooms on top of them. Most of the ground floor rooms were occupied by families, while upper rooms were rented out as embroidery and other commercial workshops.

Integrated Child Development Services (ICDS)

The cluster had only one Anganwadi, but adjoining areas had two more. When we visited, only one had children attending, but no teachers or helpers were present. We were given a number of explanations for this, but it sounded like absenteeism was a common event. This assumption was reinforced when people in the cluster said that they were unfamiliar with the whereabouts or existence of an Anganwadi. They said that no teacher or helper had ever visited their homes. The Anganwadi in which children had assembled was known and used by neighbouring residents. We were told later by a teacher from another area that the ICDS staff were on strike as they had not received their salaries for the last 3-4 months.

Drains and toilets

We spoke mostly with young mothers, who said that drains in the area had been covered around two years before. People could no longer discard household waste directly into the drains, resulting in frequent choking and overspilling. A new pay-for-use toilet block had been built around a year before. Before that the municipal toilet block had been used only by adults and children would defecate in the open or in the marshland. The women said that everyone, including children, was now using the new toilet block and open defecation had almost ceased.

Immunization

Women said that community health volunteers visited regularly to mobilise children for immunization camps and promote registration of pregnancy for hospital delivery. Community health volunteers said that they had done a lot of work on awareness over the last two years, particularly of nutrition and hygiene, but that there had been little response in terms of behaviour change. They said that they got little support from ICDS staff and that attendances at the local health post had increased over the last two years. It was unclear whether this was because more people were ill or more were seeking care.

Team members

Team members said that the cluster had many residents of southern Indian origin and commented favourably on food practices and birth spacing. They could not point to any specific changes in the last two years that would explain improvements in childhood nutrition. Some suggested that family planning and institutional delivery had increased, and childhood diarrhoea had become less common.
